# Individual and interpersonal correlates of cardiorespiratory fitness in adults – Findings from the German Health Interview and Examination Survey

**DOI:** 10.1038/s41598-019-56698-z

**Published:** 2020-01-16

**Authors:** Johannes Zeiher, Kristin Manz, Benjamin Kuntz, Nita Perumal, Thomas Keil, Gert B. M. Mensink, Jonas D. Finger

**Affiliations:** 1 Department of Epidemiology and Health Monitoring, Robert Koch Institute, Berlin, Germany; 2 Department of Infectious Disease Epidemiology, Robert Koch Institute, Berlin, Germany; 3 Institute for Clinical Epidemiology and Biometry, University of Würzburg, Würzburg, Germany; 4 Institute for Health Resort Medicine and Health Promotion, Bavarian Health and Food Safety Authority, Bad Kissingen, Germany; 5 Institute for Social Medicine, Epidemiology and Health Economics, Charité - Universitätsmedizin Berlin, Berlin, Germany

**Keywords:** Epidemiology, Risk factors

## Abstract

Cardiorespiratory fitness (CRF) is an established predictor of adverse health outcomes. The aim of this study is to investigate potential behavioral, interpersonal and socioeconomic correlates of CRF among men and women living in Germany using data from a population-based nationwide cross-sectional study. 1,439 men and 1,486 women aged 18–64 participated in the German Health Interview and Examination Survey (2008–2011) and completed a standardized sub-maximal cycle ergometer test. Maximal oxygen consumption ($\dot{V}{O}_{2}max$) in ml·min^−1^·kg^−1^ was estimated. Mean values of VO_2_max for various anthropometric, behavioral, interpersonal, and sociodemographic variables were estimated. Linear regression analyses using multiple imputations technique for missing values was performed to analyze the influence of potential correlates on CRF. Women with high alcohol consumption had higher $\dot{V}{O}_{2}max$, (*β* = 2.20; 95% CI 0.98 to 3.42) than women with low alcohol consumption and women with high occupational status had higher $\dot{V}{O}_{2}max$ (*β* = 1.83; 95% CI 0.21 to 3.44) in comparison to women with low occupational status. Among men, high fruit intake (*β* = 1.52; 95% CI 0.63 to 2.40), compared to low or medium fruit intake and performing at least 2.5 hours of total PA per week (β = 2.19; 95% CI 1.11 to 3.28), compared to less than 2.5 hours was associated with higher $\dot{V}{O}_{2}max$. Among both men and women, lower body mass index, lower waist circumference and higher levels of physical exercise were considerably associated with higher $\dot{V}{O}_{2}max$. Among women, those in higher age groups showed a considerably lower level of $\dot{V}{O}_{2}max$ compared with those aged 18–24. Furthermore, mean estimated $\dot{V}{O}_{2}max$ was higher among men (36.5; 95% CI 36.0 to 37.0) than among women (30.3; 95% CI 29.8 to 30.7). Despite the cross-sectional nature of the current study, we conclude that several behavioral, anthropometric, and sociodemographic factors are associated with CRF in the general adult population in Germany. These results can provide evidence to tailor prevention measures according to the needs of specific subgroups.

## Introduction

**Figure 1:**
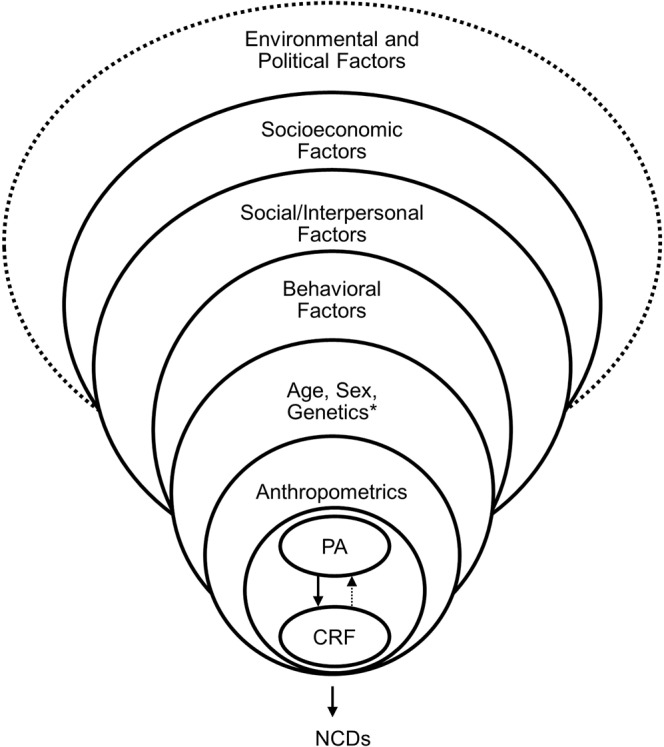
Schematic conceptual framework of the correlates of cardiorespiratory fitness (adapted from^5^). Solid lines: potential domains of the correlates of cardiorespiratory fitness investigated in the present study. Dotted lines: potential domains of the correlates of cardiorespiratory fitness *not* investigated in the present study. *Genetic factors were *not* investigated in the present study. *PA* physical activity, *CRF* cardiorespiratory fitness, *NCDs* non-communicable diseases.

Cardiorespiratory fitness (CRF) is an important marker of cardiovascular health and thus a crucial factor in the prevention of non-communicable diseases. CRF, defined as the ability of circulatory, respiratory and muscular systems to supply oxygen during prolonged physical exercise^1^, has a strong inverse relation to the incidence cardiovascular diseases^2^, cancer^3^, diabetes mellitus, depression^4^ and all-cause mortality^2^. Taking into account the impact of CRF on individual health, efforts should be taken to enhance fitness in the general population. For the development of adequate interventions, knowledge about the causes of CRF, as well as population groups at elevated risk of having a low CRF, is crucial. Figure 1 shows a conceptual framework of the potential correlates of CRF adapted from a model proposed by Després^5^. Although CRF is partly genetically determined^6^, it can be enhanced by regular endurance exercise^7^, and further factors may play a role^8^. CRF has been shown to decrease with age^9,10^ and is on average lower among women than men^11^. Furthermore, numerous studies have demonstrated an association between anthropometric measures, such as waist circumference (WC) or body mass index (BMI), with CRF^8^. Following explanatory ecological models on physical activity (PA)^12,13^, one can postulate that further determinants and correlates of CRF on the individual, interpersonal, socioeconomic or environmental level could exist^5,8,14^. In fact, CRF has been linked to behavioral (e.g., alcohol consumption^15^), socioeconomic (e.g., education^16^) and environmental factors (e.g., commuting distance^17^). Finally, all of these factors are influenced by an environmental and political framework.

However, evidence of consistent associations between CRF and many of these factors is limited^8^. While basic sociodemographic factors such as age and sex as well as physical activity and anthropometric factors have been investigated in multiple settings, research on other health behaviors or interpersonal factors is scarce. Furthermore, to our knowledge, no study has yet examined potential influencing factors of CRF within the German general population. We therefore aimed to investigate potential behavioral, interpersonal and socioeconomic correlates of CRF among men and women living in Germany using data from a population-based nationwide cross-sectional study.

## Methods

### Study design

The present analysis uses cross-sectional data from the German Health Interview and Examination Survey for Adults (DEGS1). DEGS1, a nationwide population based health examination survey, is part of the Federal Health Monitoring System operated by the Robert Koch Institute^18^. The study design is described in detail elsewhere^19^. Briefly, 7,238 individuals aged 18 to 79 years participated in the physical measurements component of the DEGS1. The survey design is based on a two-stage cluster random sampling procedure. In the first step, 180 sample points were randomly selected and stratified to represent regional distributions. In the second step, within these 180 units, adults were randomly drawn from local population registries stratified by 10-year age groups. Data collection took place between November 2008 and December 2011. The response rate was 42%. All methods were performed in accordance with the relevant guidelines and regulations.

Of the total sample of 5,262 individuals aged 18 to 64 years, 3,110 subjects were categorized as test-qualified for the cycle ergometer test test. Overall, 3,030 participants completed the exercise test (97.4%). $\dot{V}{O}_{2}max$ was estimated for all participants reaching at least 75% of the age-predicted maximum heart rate. 204 (6.7%) of the participants terminated the test before reaching this heart rate. As a result, the final study sample comprised of 2,826 participants, 1,447 of whom were women and 1,379 were men (see flow diagram of participants; Supplementary Fig. 1, Additional File 1).

### Outcome variable: cardiorespiratory fitness

CRF was measured in participants aged 18–64 years using a standardized, submaximal cycle ergometer test (Ergosana Sana Bike 350/450, Ergosana, Bitz, Germany). Test methodology, test protocol, and exclusion criteria are described in detail elsewhere^11,20^. The participants initially completed a modified version of the Physical Activity Readiness-Questionnaire (PAR-Q)^21,22^. In participants with contradictions reported according to PAR-Q, a physician decided whether or not such participants should be enrolled into the exercise test. CRF was assessed using the test protocol recommended by the World Health Organization (WHO)^23^: Beginning at 25 watts, the workload was incrementally increased by 25 watts every two minutes until 85% of the estimated age-specific maximal heartrate was exceeded, a maximum level of 350 watts was achieved or the test personnel terminated the test. Heart rate was monitored continuously throughout the test. The formula *208–0.7 · Age* was used to calculate the age-predicted maximum heart rate (HR_max_)^24^. To derive physical work capacity at HR_max_ (PWC_100%_), the measured heart rate (beats per minute) during the incremental phase was regressed against corresponding workload in watts for each participant. Assuming a linear relationship between heart rate and workload, PWC_100%_ was obtained by extrapolation using the individual regression equation *PWC*_*100%*_ = *intercept* + *HR*_*max*_ *· slope*^25^. PWC_100%_ was further converted to $\dot{V}{O}_{2}max$ using a metabolic equation provided by the American College of Sports Medicine^26^: *3.5* *ml·min*^−*1*^*·kg*^−*1*^ + *12.24·(PWC*_*100%*_)*·(body weight*^*−1*^*)*.

### Potential correlates of cardiorespiratory fitness

A comprehensive systematic literature review was performed in order to identify potential individual and socioeconomic correlates of CRF^8,14,16^. Potential interpersonal correlates of CRF were derived from evidence regarding the association of these factors and PA^12,27,28^. Based on this evidence, we developed a conceptual framework that depicts potential interrelations (Fig. 1,^8^). Corresponding covariates described below were then selected out of the DEGS1 variable list. Information on these covariates in the DEGS1 was assessed with self-administered questionnaires, physical examinations or tests by trained study personnel following standardized procedures^19^.

#### Behavioral factors

Smoking status was classified as current (including occasional smoking), ex- or never smoking. A self-administered food frequency questionnaire was used to measure intake frequency and portion size in the last four weeks for a total of 53 food and beverage groups. This food frequency questionnaire was validated and showed reasonable validity against two 24-hour recalls^29^. We selected specific food-groups distinguishing between health enhancing (“fruits” and “vegetables”) and health compromising products (“sugar rich drinks”, “sugar rich foods” and “junk foods”) based on evidence from the literature^30^. Quantities of intake of the food-groups were calculated by combining the frequency of intake and the portion size of the relevant food and beverage groups, and classifying them into two categories using sex-specific quintiles: low to moderate intake (quintile 1–3) and high intake (quintile 4–5). A detailed flowchart of food group selection and categorization can be found in Supplementary Fig. 2, Additional File 1. Ethanol in grams per day was estimated by multiplying the calculated quantity of each alcoholic beverage with standard ethanol content. Cumulated consumption was classified as low alcohol consumption (quintile 1), medium alcohol consumption (quintile 2–4), and high alcohol consumption (quintile 5) using sex-specific quintiles (Supplementary Fig. 2, Additional File 1).

#### Socioeconomic factors

Participants’ need-weighted household net income (net equivalent income) was calculated based on information about estimated net income per month and number of individuals living in the household^31^. Income was then grouped into three categories: below 60%, 60–150% and above 150% of the median net household equivalent income, representing an income below the relative poverty line and an intermediate or relatively high income, respectively^32^. Educational level was assessed using the ‘Comparative Analysis of Social Mobility in Industrial Nations’ (CASMIN)^33^ and classified into three categories (primary, secondary, and tertiary education). Occupational status was determined using the International Socio-Economic Index of Occupational Status (ISEI)^34^ based on current occupation of the participants. The variable was classified into three groups: low (quintile 1), medium (quintile 2–4), high occupational status (quintile 5). Participants were further asked if they were born in Germany or abroad.

#### Interpersonal factors

Social support was assessed using the Oslo Three-Item Social Support Scale (OSS-3)^35^ and classified as poor (3–8), moderate (9–11), and strong (12–14) social support. Marital status was grouped as single, married (while living together), and separated/divorced/widowed.

#### Anthropometric factors

Body weight and height was measured using portable electronic scales (SECA, Germany) and stadiometer (Holtain, UK). BMI (kg/m^2^) was categorized according to the WHO guidelines^36^ into underweight (BMI <18.5), normal weight (18.5≤ BMI <25), overweight (25 ≤ BMI <30) and obese (BMI ≥30). WC was measured at the smallest site between the lowest rib and the superior border of the iliac crest with flexible, non-stretchable measurement tape^37^. WC was categorized as ‘normal’, ‘increased’ and ‘strongly increased’ according to international guidelines^38^.

#### Physical activity-related factors

Total PA was assessed by asking participants the number of days in an average week where they were physically active enough to start sweating or get out of breath. If they reported any PA, they were further asked about the duration of PA on such days^39^. Based on this information participants were classified into 2 groups, using the WHO recommendation as cut-off: <2.5 hours per week and ≥2.5 hours per week. Participants were asked “How often do you engage in physical exercise?”^39^, with responses categorized into three groups: no physical exercise, <2 hours/week, ≥2 hours/week.

### Statistical analyses

All statistical analyses were performed with Stata 15.1 (Stata Corp., College Station, TX, USA). Stata survey commands were used to adequately account for the cluster sampling design when calculating confidence intervals. Weighting factors were used, unless otherwise noted, to adjust the distribution of the sample to match those of the German population by sex, age, education and region for all calculations^40^. Scatterplots were computed to show the crude, unweighted association between age, WC and BMI with $\dot{V}{O}_{2}max$. Fractional-polynomial prediction plots with 95%-confidence intervals (95% CI) were then fitted to show the estimated associations between these variables. Mean $\dot{V}{O}_{2}max$ with 95% CI was calculated by behavioral, sociodemographic and interpersonal, anthropometric, and PA indicators. Multivariable linear regression models were computed to estimate the associations between potential correlates and estimated $\dot{V}{O}_{2}max$, stratified by sex. In Model 1 only age and behavioral factors (without total PA/ physical exercise) were included. In the next model (Model 2), sociodemographic and interpersonal factors were added. The subsequent models included the anthropometric (Model 3) and PA-related factors (Model 4). A complete case analysis would have led to a considerably reduced and less representative sample (n = 573 with missing values in at least one covariate; 20.3% of eligible cases [see Supplementary Fig. 1, Additional File 1]). Thus, we conducted multiple missing values imputation using chained equations^41^ for BMI, WC, occupational status, education, migration status, marital status, total PA, physical exercise, smoking status, alcohol consumption as well as all food variables. We imputed 30 sex-specific datasets. Linear regression analyses were performed with each of the 30 datasets and the final coefficients are the results from all datasets combined. Multivariable linear regressions were performed using Stata multiple imputation commands in combination with the survey commands.

### Ethics approval and consent to participate

The study protocol was approved by the Federal and State Commissioners for Data Protection and by the ethics committee of the Charité-University Medicine Berlin (No. EA2/047/08). Informed written consent was obtained from all participants.

## Results

Overall, 47.4% of the included survey participants were women and the mean age of all participants was 38.4 years (95% CI: 37.9 to 38.8). CRF test participants were younger, not retired, higher educated, and reported higher levels of physical exercise than individuals who were not qualified for the test (Supplementary Table 1, Additional File 1).

**Figure 2:**
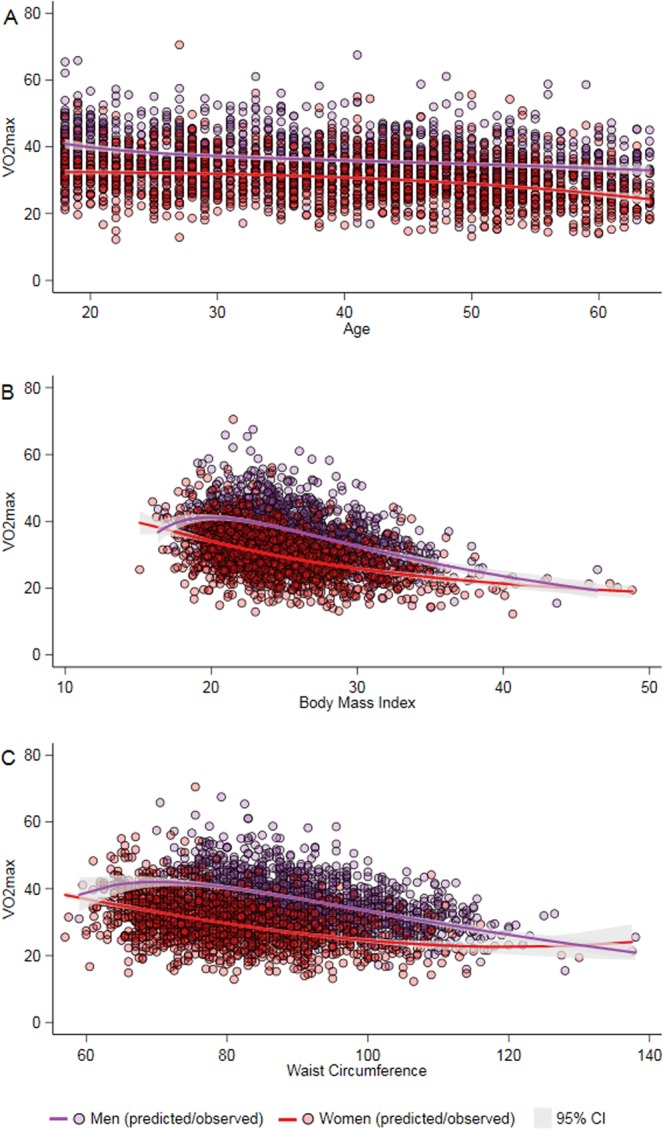
Association of (**A**) age, (**B**) body mass index and (**C**) waist circumference with cardiorespiratory fitness ($\dot{V}{O}_{2}max$) in men and women. $\dot{V}{O}_{2}max$ Maximal oxygen consumption; *CI* confidence interval.

Figure 2 shows the crude and fitted association between age, BMI and WC with estimated $\dot{V}{O}_{2}max$, indicating clear inverse associations between age, BMI, and WC with estimated $\dot{V}{O}_{2}max$.

### Mean $\dot{{\boldsymbol{V}}}$*O*_2_*max*

Table 1 presents mean $\dot{V}{O}_{2}max$ by covariates selected for this study. Mean $\dot{V}{O}_{2}max$ (in ml·min^−1^·kg^−1^) was higher among men (36.5; 95% CI 36.0 to 37.0) than among women (30.3; 95% CI 29.8 to 30.7). $\dot{V}{O}_{2}max$ decreased with age in both women and men.

**Table 1:** Bivariate associations between $\dot{V}{O}_{2}max$ and potential correlates.

	Men		Women		Total	
	Mean $\dot{{\boldsymbol{V}}}{{\boldsymbol{O}}}_{{\bf{2}}}{\boldsymbol{m}}{\boldsymbol{a}}{\boldsymbol{x}}$	(95% CI)	Mean $\dot{{\boldsymbol{V}}}{{\boldsymbol{O}}}_{{\bf{2}}}{\boldsymbol{m}}{\boldsymbol{a}}{\boldsymbol{x}}$	(95% CI)	Mean $\dot{{\boldsymbol{V}}}{{\boldsymbol{O}}}_{{\bf{2}}}{\boldsymbol{m}}{\boldsymbol{a}}{\boldsymbol{x}}$	(95% CI)
Total (n = 2.826)	36.5	(36.0–37.0)	30.3	(29.8–30.7)	33.6	(33.1–34.0)
**Age**						
18–24 Years (n = 444)	39.7	(38.5–40.9)	32.1	(31.1–33.1)	36.2	(35.4–37.0)
25–34 Years (n = 579)	37.6	(36.5–38.6)	32.4	(31.4–33.4)	35.2	(34.4–36.1)
35–44 Years (n = 658)	35.8	(34.8–36.8)	30.7	(29.9–31.4)	33.3	(32.7–34.0)
45–54 Years (n = 724)	34.8	(33.8–35.7)	28.8	(28.0–29.6)	31.8	(31.1–32.5)
55–64 Years (n = 421)	34.2	(33.0–35.4)	25.9	(24.9–27.0)	30.1	(29.2–31.1)
Missing = 0						
**Waist circumference**						
Normal (n = 1,661)	38.8	(38.2–39.4)	32.4	(31.8–33.0)	35.9	(35.4–36.4)
Increased (n = 595)	34.3	(33.4–35.3)	28.7	(27.8–29.6)	31.4	(30.7–32.1)
Strongly increased (n = 568)	30.6	(29.6–31.6)	25.7	(25.0–26.4)	28.2	(27.5–28.8)
Missing = 2						
**Body mass index**						
Underweight (n = 52)	38.7	(36.9–40.4)	36.1	(33.1–39.0)	36.8	(34.6–39.0)
Normal Weight (n = 1,420)	40.0	(39.2–40.8)	32.1	(31.6–32.7)	35.5	(35.0–36.1)
Overweight (n = 982)	35.2	(34.6–35.9)	27.9	(27.1–28.8)	32.6	(32.0–33.1)
Obese (n = 364)	30.5	(29.4–31.6)	24.4	(23.5–25.2)	28.1	(27.2–28.9)
Missing = 8						
**Smoking status**						
Daily/ Occasionally (n = 933)	36.8	(36.0–37.5)	30.4	(29.6–31.2)	34.1	(33.5–34.7)
Former (n = 732)	34.6	(33.5–35.7)	29.3	(28.5–30.2)	32.1	(31.4–32.9)
Never (n = 1,147)	37.5	(36.6–38.3)	30.8	(30.1–31.4)	33.9	(33.3–34.6)
Missing = 14						
**Alcohol consumption**						
Low (n = 501)	35.7	(34.6–36.8)	28.7	(27.8–29.7)	32.4	(31.5–33.3)
Moderate (n = 1,710)	37.0	(36.4–37.6)	30.3	(29.7–30.9)	33.8	(33.3–34.3)
High (n = 586)	36.1	(34.9–37.3)	31.8	(30.7–32.9)	34.1	(33.3–34.9)
Missing = 29						
**Sugar-rich foods intake**						
Low/moderate (n = 1,618)	36.2	(35.5–36.9)	30.1	(29.5–30.7)	33.3	(32.7–33.8)
High (n = 1.096)	37.2	(36.4–37.9)	30.6	(29.9–31.4)	34.0	(33.4–34.6)
Missing = 112						
**Sugar-rich drinks intake**						
Low/moderate (n = 1,733)	36.1	(35.5–36.7)	30.5	(29.9–31.1)	33.4	(33.0–33.9)
High (n = 1.047)	37.3	(36.6–38.1)	30.1	(29.3–30.8)	33.8	(33.2–34.4)
Missing = 46						
**Junk foods intake**						
Low/moderate (n = 1,733)	35.9	(35.3–36.5)	29.8	(29.2–30.4)	33.0	(32.5–33.5)
High (n = 994)	37.6	(36.7–38.5)	31.0	(30.2–31.8)	34.5	(33.8–35.2)
Missing = 99						
**Fruit intake**						
Low/moderate (n = 1,717)	36.2	(35.6–36.8)	30.0	(29.4–30.6)	33.4	(32.9–33.9)
High (n = 1.054)	37.4	(36.5–38.3)	30.8	(30.0–31.6)	33.9	(33.3–34.6)
Missing = 55						
**Vegetable intake**						
Low/moderate (n = 1,680)	36.3	(35.7–36.9)	30.0	(29.4–30.5)	33.3	(32.8–33.8)
High (n = 1.073)	36.9	(36.2–37.7)	30.9	(30.1–31.6)	34.1	(33.5–34.7)
Missing = 73						
**Country of birth**						
Born in Germany (n = 2,508)	36.9	(36.4–37.4)	30.5	(30.0–31.0)	33.8	(33.4–34.3)
Born outside Germany (n = 273)	34.8	(33.5–36.2)	29.0	(27.7–30.4)	32.1	(31.1–33.2)
Missing = 45						
**Educational classification**						
Primary (n = 567)	34.9	(33.9–35.9)	27.7	(26.7–28.7)	32.0	(31.0–32.9)
Secondary (n = 1,667)	37.1	(36.5–37.7)	30.5	(29.9–31.0)	33.8	(33.3–34.2)
Tertiary (n = 577)	37.0	(36.0–38.1)	32.6	(31.7–33.6)	35.0	(34.2–35.7)
Missing = 15						
**Occupational status**						
Low (Q1) (n = 485)	35.6	(34.5–36.6)	28.0	(26.9–29.2)	32.3	(31.4–33.3)
Medium (Q2-Q4) (n = 1,512)	36.5	(35.7–37.2)	29.9	(29.3–30.5)	33.1	(32.6–33.6)
High (Q5) (n = 525)	37.2	(36.2–38.3)	33.0	(31.8–34.3)	35.5	(34.6–36.3)
Missing = 304						
**Income (% of median-income)**						
<60% (n = 479)	36.6	(35.3–37.9)	29.1	(27.8–30.4)	33.2	(32.2–34.3)
60 −<150% (n = 1,690)	36.2	(35.6–36.8)	29.9	(29.3–30.5)	33.2	(32.7–33.6)
≥150% (n = 657)	37.2	(36.2–38.2)	32.3	(31.4–33.2)	34.9	(34.2–35.6)
Missing = 0						
**Social support**						
Poor support (n = 244)	36.4	(34.8–38.0)	28.9	(27.0–30.7)	33.4	(32.0–34.8)
Moderate support (n = 1,394)	36.3	(35.6–37.0)	29.6	(29.0–30.2)	33.3	(32.7–33.8)
Strong support (n = 1,171)	36.8	(35.9–37.7)	31.1	(30.5–31.8)	33.9	(33.3–34.4)
Missing = 17						
**Marital status**						
Single (n = 994)	38.2	(37.4–39.0)	31.9	(31.1–32.6)	35.6	(35.0–36.2)
Married, living together (n = 1,565)	35.0	(34.3–35.6)	29.6	(29.0–30.1)	32.2	(31.7–32.7)
Separated/Divorced/Widowed (n = 240)	36.9	(34.8–39.0)	28.8	(27.4–30.2)	32.4	(30.9–33.9)
Missing = 27						
**Total physical activity per week**						
<2.5 hours (n = 2,124)	35.2	(34.5–35.8)	29.9	(29.4–30.4)	32.5	(32.0–32.9)
≥2.5 hours (n = 643)	39.6	(38.7–40.5)	32.2	(31.2–33.3)	37.0	(36.2–37.8)
Missing = 59						
**Physical exercise per week**						
No physical exercise (n = 672)	33.0	(32.2–33.9)	27.6	(26.8–28.4)	30.4	(29.7–31.1)
<2 hours (n = 1,249)	36.1	(35.4–36.7)	30.0	(29.3–30.6)	32.9	(32.3–33.4)
≥2 hours (n = 869)	39.5	(38.5–40.4)	33.4	(32.6–34.2)	37.0	(36.4–37.7)
Missing = 36						

$\dot{V}{O}_{2}max$: maximal oxygen consumption; CI: confidence intervals.

Further descriptive binary analyses showed that mean $\dot{V}{O}_{2}max$ was higher among women with high levels of alcohol consumption, secondary or tertiary education, high occupational status, high income, being single, having normal or underweight BMI, having a normal WC, being physically active and participating in physical exercise. Among men, mean $\dot{V}{O}_{2}max$ was higher among those with high junk food intake, being born in Germany, having secondary or tertiary education, being single, having normal or underweight BMI, having a normal WC, being physically active and participating in physical exercise.

### Multivariable analyses

Multivariable analyses indicated that age, smoking, alcohol consumption, fruit intake, place of birth, WC, BMI, and physical exercise were associated with estimated $\dot{V}{O}_{2}max$ in both sexes (Table 2 and Table 3). While vegetable intake, income and occupational status were only observed to be associated with $\dot{V}{O}_{2}max$ among women, sugar-rich food intake, marital status and total PA showed a considerable association with $\dot{V}{O}_{2}max$ only among men.

**Table 2:** Correlates of $\dot{V}{O}_{2}max$ in women.

	Model 1		Model 2		Model 3		Model 4	
	${\boldsymbol{\beta }}$	(95 % CI)	${\boldsymbol{\beta }}$	(95 % CI)	${\boldsymbol{\beta }}$	(95 % CI)	${\boldsymbol{\beta }}$	(95 % CI)
**Age**								
18–24 Years	(ref.)		(ref.)		(ref.)		(ref.)	
25–34 Years	0.44	(−0.87–1.76)	−0.36	(−1.71–1.00)	0.13	(−1.10–1.36)	0.89	(−0.33–2.12)
35–44 Years	**−1.53**	**(−2.71–−0.35)**	**−2.34**	**(−3.82–−0.86)**	−1.12	(−2.55–0.31)	−0.28	(−1.64–1.08)
45–54 Years	**−3.27**	**(−4.41–−2.12)**	**−4.00**	**(−5.46–−2.53)**	**−2.79**	**(−4.27–−1.31)**	**−2.08**	**(−3.49–−0.67)**
55–64 Years	**−6.53**	**(−7.98–−5.09)**	**−7.04**	**(−8.79–−5.30)**	**−4.98**	**(−6.74–−3.22)**	**−4.27**	**(−5.94–−2.60)**
**Smoking status**								
Never	(ref.)		(ref.)		(ref.)		(ref.)	
Daily/Occasionally	−0.89	(−1.93–0.15)	−0.61	(−1.66–0.44)	−0.15	(−1.11–0.81)	0.12	(−0.80–1.04)
Former	−0.85	(−1.83–0.12)	**−1.01**	**(−1.95–−0.06)**	−0.54	(−1.45–0.36)	−0.67	(−1.55–0.20)
**Alcohol consumption**								
Low	(ref.)		(ref.)		(ref.)		(ref.)	
Moderate	**1.88**	**(0.86–2.90)**	**1.11**	**(0.13–2.09)**	0.81	(−0.09–1.72)	0.75	(−0.13–1.62)
High	**3.69**	**(2.27–5.11)**	**2.79**	**(1.46–4.12)**	**2.42**	**(1.13–3.71)**	**2.20**	**(0.98–3.42)**
**Sugar-rich foods intake**								
Low/moderate	(ref.)		(ref.)		(ref.)		(ref.)	
High	0.75	(−0.16–1.66)	0.71	(−0.17–1.59)	0.57	(−0.24–1.37)	0.44	(−0.34–1.22)
**Sugar-rich drinks intake**								
Low/moderate	(ref.)		(ref.)		(ref.)		(ref.)	
High	−0.85	(−1.78–0.08)	−0.68	(−1.56–0.20)	−0.75	(−1.56–0.05)	−0.73	(−1.48–0.03)
**Junk foods intake**								
Low/moderate	(ref.)		(ref.)		(ref.)		(ref.)	
High	0.080	(−0.89–1.05)	0.23	(−0.63–1.10)	0.52	(−0.29–1.32)	0.75	(−0.01–1.51)
**Fruit intake**								
Low/moderate	(ref.)		(ref.)		(ref.)		(ref.)	
High	**1.04**	**(0.04–2.04)**	**0.91**	**(0.004–1.81)**	**1.06**	**(0.30–1.82)**	0.65	(−0.08–1.37)
**Vegetable intake**								
Low/moderate	(ref.)		(ref.)		(ref.)		(ref.)	
High	**0.82**	**(0.02–1.63)**	**0.79**	**(0.03–1.55)**	**0.96**	**(0.28–1.65)**	0.62	(−0.06–1.30)
**Place of birth**								
Born in Germany			(ref.)		(ref.)		(ref.)	
Born outside Germany			**−2.19**	**(−3.59–−0.79)**	**−2.01**	**(−3.42–−0.60)**	−1.34	(−2.70–0.03)
**Education**								
Primary			(ref.)		(ref.)		(ref.)	
Secondary			0.89	(−0.15–1.92)	0.59	(−0.33–1.52)	0.80	(−0.12–1.71)
Tertiary			1.46	(−0.11–3.02)	0.78	(−0.76–2.32)	0.90	(−0.58–2.37)
**Occupational status**								
Low			(ref.)		(ref.)		(ref.)	
Medium			**1.46**	**(0.25–2.67)**	**1.28**	**(0.13–2.43)**	0.94	(−0.15–2.02)
High			**2.76**	**(0.97–4.55)**	**2.42**	**(0.67–4.18)**	**1.83**	**(0.21–3.44)**
**Income (% of median income)**								
<60 %			(ref.)		(ref.)		(ref.)	
60 to <150%			0.82	(−0.54–2.19)	0.67	(−0.63–1.97)	0.52	(−0.75–1.79)
>=150%			**2.40**	**(0.66–4.15)**	1.57	(−0.09–3.22)	1.24	(−0.37–2.86)
**Social support**								
Poor			(ref.)		(ref.)		(ref.)	
Moderate			0.13	(−1.67–1.94)	−0.33	(−1.93–1.27)	−0.50	(−2.03–1.03)
Strong			0.79	(−0.99–2.58)	0.58	(−0.95–2.12)	0.08	(−1.42–1.58)
**Marital status**								
Married, living together			(ref.)		(ref.)		(ref.)	
Single			−0.33	(−1.54–0.88)	−0.59	(−1.77–0.58)	−0.59	(−1.72–0.55)
Separated/Divorced/Widowed			−0.02	(−1.33–1.29)	−0.38	(−1.69–0.92)	−0.31	(−1.53–0.91)
**Waist circumference**								
Normal					(ref.)		(ref.)	
Increased					**−1.73**	**(−2.66–−0.79)**	**−1.56**	**(−2.45–−0.68)**
Strongly increased					**−1.83**	**(−3.11–−0.54)**	**−1.61**	**(−2.85–−0.38)**
**Body mass index**								
Underweight					**2.95**	**(0.38–5.52)**	**3.13**	**(0.58–5.69)**
Normal Weight					(ref.)		(ref.)	
Overweight					**−2.21**	**(−3.18–−1.24)**	**−2.36**	**(−3.26–−1.46)**
Obese					**−5.14**	**(−6.43–−3.85)**	**−4.88**	**(−6.19–−3.57)**
**Total physical activity per week**								
<2.5 hours							(ref.)	
≥2.5 hours							0.45	(−0.53–1.43)
**Physical exercise per week**								
No physical exercise							(ref.)	
<2 hours							**1.68**	**(0.84–2.52)**
≥2 hours							**4.20**	**(3.10–5.30)**
Constant	**30.0**	**(28.5–31.6)**	**28.0**	**(25.1–30.9)**	**29.8**	**(27.2–32.5)**	**27.9**	**(25.2–30.6)**
N	1,447		1,447		1,447		1,447	
**R**^**2**^	0.136		0.199		0.310		0.356	

Coefficients and 95 % CI and shown in bold: 95 % CI does not include 0. $\dot{V}{O}_{2}max$: maximal oxygen consumption; $\beta$: linear regression coefficient; CI: confidence intervals.

**Table 3:** Correlates of $\dot{V}{O}_{2}max$ in men.

	Model 1		Model 2		Model 3		Model 4	
	${\boldsymbol{\beta }}$	(95 % CI)	${\boldsymbol{\beta }}$	(95 % CI)	${\boldsymbol{\beta }}$	(95 % CI)	${\boldsymbol{\beta }}$	(95 % CI)
**Age**								
18–24 Years	(ref.)		(ref.)		(ref.)		(ref.)	
25–34 Years	**−1.96**	**(−3.52–−0.41)**	**−1.84**	**(−3.45–−0.23)**	−0.46	(−1.94–1.01)	0.37	(−1.06–1.80)
35–44 Years	**−3.74**	**(−5.28–−2.21)**	**−3.32**	**(−5.23–−1.41)**	−1.17	(−2.96–0.63)	−0.10	(−1.86–1.66)
45–54 Years	**−4.59**	**(−6.09–−3.08)**	**−4.09**	**(−6.12–−2.05)**	**−1.94**	**(−3.80–−0.08)**	−0.96	(−2.80–0.87)
55–64 Years	**−5.37**	**(−7.12–−3.62)**	**−4.99**	**(−7.30–−2.67)**	−2.09	(−4.27–0.08)	−1.39	(−3.50–0.72)
**Smoking status**								
Never	(ref.)		(ref.)		(ref.)		(ref.)	
Daily/Occasionally	−0.61	(−1.73–0.50)	−0.35	(−1.52–0.82)	0.027	(−1.04–1.09)	0.38	(−0.62–1.38)
Former	**−2.14**	**(−3.55–−0.73)**	**−1.85**	**(−3.22–−0.49)**	−0.79	(−2.01–0.44)	−0.57	(−1.80–0.65)
**Alcohol consumption**								
Low	(ref.)		(ref.)		(ref.)		(ref.)	
Moderate	**1.48**	**(0.29–2.67)**	0.95	(−0.20–2.11)	0.80	(−0.23–1.83)	0.86	(−0.13–1.86)
High	1.34	(−0.27–2.94)	0.92	(−0.72–2.57)	0.80	(−0.60–2.20)	0.96	(−0.37–2.29)
**Sugar-rich foods intake**								
Low/moderate	(ref.)		(ref.)		(ref.)		(ref.)	
High	0.91	(−0.12–1.94)	**1.02**	**(0.01–2.03)**	0.74	(−0.19–1.66)	0.66	(−0.18–1.51)
**Sugar-rich drinks intake**								
Low/moderate	(ref.)		(ref.)		(ref.)		(ref.)	
High	0.30	(−0.65–1.25)	0.30	(−0.67–1.27)	0.65	(−0.23–1.52)	0.35	(−0.52–1.21)
**Junk foods intake**								
Low/moderate	(ref.)		(ref.)		(ref.)		(ref.)	
High	−0.001	(−1.20–1.20)	0.042	(−1.12–1.20)	0.23	(−0.78–1.25)	0.23	(−0.73–1.19)
**Fruit intake**								
Low/moderate	(ref.)		(ref.)		(ref.)		(ref.)	
High	**1.77**	**(0.73–2.81)**	**1.84**	**(0.81–2.88)**	**1.77**	**(0.82–2.72)**	**1.52**	**(0.63–2.40)**
**Vegetable intake**								
Low/moderate	(ref.)		(ref.)		(ref.)		(ref.)	
High	0.38	(−0.52–1.29)	0.15	(−0.73–1.03)	0.43	(−0.38–1.25)	0.36	(−0.43–1.15)
**Place of birth**								
Born in Germany			(ref.)		(ref.)		(ref.)	
Born outside Germany			**−1.70**	**(−3.29–−0.12)**	−1.35	(−2.71–0.01)	−1.28	(−2.59–0.04)
**Education**								
Primary			(ref.)		(ref.)		(ref.)	
Secondary			0.65	(−0.62–1.93)	0.65	(−0.50–1.80)	0.35	(−0.71–1.42)
Tertiary			1.20	(−0.72–3.12)	0.19	(−1.49–1.88)	−0.076	(−1.63–1.48)
**Occupational status**								
Low			(ref.)		(ref.)		(ref.)	
Medium			0.71	(−0.64–2.05)	0.31	(−0.87–1.48)	0.16	(−0.98–1.30)
High			0.90	(−0.98–2.77)	0.53	(−1.06–2.11)	0.31	(−1.22–1.84)
**Income (% of median income)**								
<60 %			(ref.)		(ref.)		(ref.)	
60 to <150%			−0.15	(−1.54–1.25)	−0.18	(−1.45–1.09)	−0.42	(−1.64–0.80)
>=150%			0.42	(−1.21–2.04)	0.52	(−0.91–1.94)	0.11	(−1.26–1.49)
**Social support**								
Poor			(ref.)		(ref.)		(ref.)	
Moderate			−0.12	(−1.76–1.52)	−0.76	(−2.11–0.59)	−0.61	(−1.84–0.61)
Strong			0.073	(−1.65–1.80)	0.055	(−1.41–1.52)	−0.10	(−1.45–1.25)
**Marital status**								
Married, living together			(ref.)		(ref.)		(ref.)	
Single			0.97	(−0.48–2.43)	0.04	(−1.33–1.41)	−0.34	(−1.67–0.99)
Separated/Divorced/Widowed			**2.34**	**(0.42–4.26)**	1.38	(−0.48–3.25)	0.98	(−0.76–2.72)
**Waist circumference**								
Normal					(ref.)		(ref.)	
Increased					**−2.08**	**(−3.26–−0.91)**	**−1.58**	**(−2.71–−0.45)**
Strongly increased					**−4.04**	**(−5.42–−2.67)**	**−2.92**	**(−4.23–−1.60)**
**Body mass index**								
Underweight					−1.14	(−3.28–1.01)	−0.84	(−3.26–1.59)
Normal Weight					(ref.)		(ref.)	
Overweight					**−2.90**	**(−3.95–−1.84)**	**−3.00**	**(−4.00–−1.99)**
Obese					**−5.35**	**(−7.01–−3.69)**	**−5.79**	**(−7.39–−4.20)**
**Total physical activity per week**								
<2.5 hours							(ref.)	
≥2.5 hours							**2.19**	**(1.11–3.28)**
**Physical exercise per week**								
No physical exercise							(ref.)	
<2 hours							**1.99**	**(1.00–2.98)**
≥2 hours							**3.74**	**(2.59–4.88)**
Constant	**38.0**	**(36.2–39.7)**	**36.5**	**(33.2–39.7)**	**38.7**	**(35.7–41.7)**	**35.7**	**(32.8–38.6)**
N	1,379		1,379		1,379		1,379	
R^2^	0.098		0.123		0.279		0.341	

Coefficients and 95% CI and shown in bold: 95% CI does not include 0. $\dot{V}{O}_{2}max$: maximal oxygen consumption; $\beta$: linear regression coefficient; CI: confidence intervals.

The fully adjusted Model 4 showed a considerably lower level of $\dot{V}{O}_{2}max$ for women in higher age groups compared with those aged 18–24: among women aged 45 to 54 years, $\dot{V}{O}_{2}max$ decreased by $\beta$ = −2.08 (95% CI −3.49 to −0.67) and in women aged 55 to 64 years by ($\beta$ = −4.27; 95% CI −5.94 to −2.60), respectively (Table 2). Women with high alcohol consumption had higher $\dot{V}{O}_{2}max$, ($\beta$ = 2.20; 95% CI 0.98 to 3.42) than women with low alcohol consumption. Similarly, women with high occupational status had higher $\dot{V}{O}_{2}max$ ($\beta$ = 1.83; 95% CI 0.21 to 3.44) in comparison to women with low occupational status and those with increased and strongly increased WC had lower $\dot{V}{O}_{2}max$ than those with normal WC (increased WC: $\beta$ = −1.56; 95% CI −2.45 to −0.68, and strongly increased WC: $\beta$ = −1.61; 95% CI −2.85 to −0.38). In addition, an inverse association was observed between BMI and $\dot{V}{O}_{2}max$ among women: while underweight women had higher $\dot{V}{O}_{2}max$ compared to normal-weight women ($\beta$ = 3.13; 95% CI 0.58 to 5.69), overweight ($\beta$ = −2.36; 95% CI −3.26 to −1.46) and obese ($\beta$ = −4.88; 95% CI −6.19 to −3.57) women showed considerably lower $\dot{V}{O}_{2}max$ compared to normal-weight women. Furthermore, among women $\dot{V}{O}_{2}max$ increased with the amount of physical exercise per week, with $\beta$ = 1.68 (95% CI 0.84 to 2.52) for up to two hours and $\beta$ = 4.20 (95% CI 3.10 to 5.30) for more than two hours of physical exercise per week compared to women not engaging in any physical exercise.

Among men high fruit intake was associated with higher $\dot{V}{O}_{2}max$, ($\beta$ = 1.52; 95% CI 0.63 to 2.40), compared to low or medium fruit intake (Table 3). As among women, $\dot{V}{O}_{2}max$ was lower among men with increased WC ($\beta$ = −1.58; 95% CI −2.71 to −0.45) and strongly increased WC ($\beta$ = −2.92; 95% CI −4.23 to −1.60) in comparison to men with normal WC. Overweight ($\beta$ = −3.00; 95% CI −4.00 to −1.99) and obese ($\beta$ = −5.79; 95% CI −7.39 to −4.20) men had lower $\dot{V}{O}_{2}max$ compared to men with normal weight. Both total PA and physical exercise were considerably associated with $\dot{V}{O}_{2}max$ among men. Men who met the WHO PA recommendation of at least 2.5 hours of total PA per week showed higher $\dot{V}{O}_{2}max$ ($\beta$ = 2.19; 95% CI 1.11 to 3.28) than men who did not meet the PA recommendation. An increasing level of $\dot{V}{O}_{2}max$ was also associated with increasing weekly hours of physical exercise participation: men with up to two hours of physical exercise per week, ($\beta$ = 1.99; 95% CI 1.00 to 2.98), and men with two hours or more of physical exercise per week ($\beta$ = 3.74; 95% CI 2.59 to 4.88) showed higher $\dot{V}{O}_{2}max$ compared to men who did not engage in any physical exercise.

### Model comparison and additional analyses

Explained variance (R^2^) increased from 13.6% in Model 1 to 35.6% in Model 4 for women and from 9.8% to 34.1% for men. Age was negatively associated with $\dot{V}{O}_{2}max$ among both sexes and indicated a strong effect size in Model 1 and Model 2. After adjustment for BMI and WC (Model 3), the effect size of age decreased for both sexes, but more strongly for men than for women. The coefficients of behavioral, interpersonal and socioeconomic factors slightly decreased after additional adjustments but the associations remained relatively stable overall. Among women, the effect size of high income on $\dot{V}{O}_{2}max$ became smaller after adjustment for BMI and WC (Model 3) and the effect sizes of fruit intake, vegetable intake and of being born outside Germany all became smaller after adjustment for PA-related factors (Model 4). Among men, the effects of being divorced, separated or widowed and being a former smoker decreased after adjustment for anthropometric measures (Model 3). After adjustment for PA-related factors (Model 4), coefficients remained relatively stable.

As additional analyses the final Model 4 for the non sex-stratified full sample using sex as an additional covariate was computed (Supplementary Table 2, Additional File 1). Even after full adjustment women showed lower levels of estimated $\dot{V}{O}_{2}max$ than men ($\beta$ = −6.56; 95% CI (−7.17 to −5.94)). Furthermore, we conducted a sensitivity analysis and compared the final imputed model with a complete-case model without imputation of missing values: Despite slightly wider confidence intervals, only small deviations among the coefficients appeared (see Supplementary Figs. 3, 4, Additional File 1).

## Discussion

In this study we were able to replicate the well-established relationships in the literature between anthropometric measures (BMI and WC), total PA and physical exercise, and estimated $\dot{V}{O}_{2}max$ using data from a nation-wide, population-based cross-sectional health examination survey among adults in Germany. In addition, we demonstrated associations between a range of additional individual and interpersonal factors and CRF. Among women, high levels of alcohol consumption, high occupational status, lower BMI, smaller WC and higher physical exercise level were associated with higher $\dot{V}{O}_{2}max$. Among men, lower age, high intake of fruits, lower BMI, smaller WC, at least 2.5 hours of PA per week and higher physical exercise level were associated with higher $\dot{V}{O}_{2}max$.

### Sex and age differences

The observation that men have a higher CRF than women has been reported in a number of previous studies, both internationally and in Germany^8,11,42,43^. In the current study, women had 17% lower $\dot{V}{O}_{2}max$ than men, which is comparable to an often reported sex difference in CRF of about 20%^8,11^. Lower fitness among women compared to men is commonly explained by women’s smaller organ and body size and higher percentage of body fat on average and lower skeletal muscle mass^7,44^. Additional analyses with sex as an additional covariate showed that sex differences are not mediated by the anthropometric, behavioral, sociodemographic and interpersonal factors used in the fully adjusted model.

Our finding of decreasing $\dot{V}{O}_{2}max$ with increasing age corresponds with evidence from both cross-sectional and cohort studies^8–10^. Potential explanations are physiological adjustments during the aging process, such as muscle mass atrophy, increasing burden of disease, and onset of physical limitations. Although, the use of coronary drugs and cardiovascular diseases were contraindications for test participation in this study, other illnesses and medications could affect the results^20^. Therefore, our study-sample consists of a relatively healthy population aged <65 years.

After adjustment for total PA and physical exercise (Model 4), there was no considerable age-effect among men. According to the literature, the effect of PA on the decline in CRF over the life course is inconclusive^45,46^. While longitudinal studies found that individuals with enhanced PA levels had a smaller decline in CRF than sedentary individuals^46^, there was no evidence for the mitigation of the effect by PA in meta-analyses of cross-sectional data^47,48^.

### Behavioral factors

Former smokers demonstrated lower fitness compared to non-smokers in bivariate analyses and Model 2, but the effect decreased when controlling for anthropometric and PA-related factors. Most studies investigating the association between smoking and CRF have found lower fitness levels among smokers compared with non-smokers, but some other studies have not found such association^8^. Two studies with NHANES data, adjusted for multiple variables, even observed higher fitness levels among young to middle-aged adult current smokers in both sexes^49^ or in the male subsample^50^. While all studies observing no or a positive association had a cross sectional design, all longitudinal studies observed lower CRF levels among smokers compared with non-smokers^51–55^. Thus, in a cross-sectional study design, the effect of smoking on CRF might be hidden due to confounding, e.g. by age, as especially ex-smokers are usually older than current or never smokers. They may also have quit smoking because of health problems. In our analysis, the adequate elucidation of the effect of smoking on CRF could be hampered by the use of smoking status instead of quantitative measures of smoking (e.g., pack years).

We observed higher CRF among women with high levels of alcohol consumption. A study investigating the association between alcohol consumption and CRF based on five independent population-based studies from the US and Germany (including DEGS1) found an inverse u-shaped association with higher fitness levels among moderately drinking men and women^15^. However, these findings are in line with the results of our study, as Baumeister *et al*. observed a maximum of the curve at a very high level of consumption among women (ca. 35 g/d). In DEGS1, few women (<2%) reach this high level of consumption and correspondingly most women in the high consumption category consume less alcohol per day. Higher levels of fitness among individuals who consume alcohol are consistent with research on PA and alcohol intake. Studies in the past found that moderate or even high alcohol consumption is associated with higher levels of PA^56^. However, the mechanisms behind this relation are not fully understood. One possible explanation is that both PA and alcohol consumption work as rewarding stimuli and have overlapping effects in individuals stress regulation mechanisms^56^. Another possible explanation could be that specific personality characteristics like extroversion might correlate with both alcohol consumption (opportunities) and physical exercise (with others). Finally, confounding has to be considered as a possible explanation, as alcohol consumption is more common among higher educated women in Germany^57,58^ who are practicing a lifestyle that includes more physical exercise^39,59^ translating into higher CRF.

We observed higher CRF among men with high fruit intake. This is in line with results from the CARDIA-Study, where higher CRF was observed among men with a relative high level of fruit and vegetable intake^60^. Although in the final model of our study none of the other food groups (sugar-rich foods, sugar-rich drinks, junk food, vegetables) showed association with $\dot{V}{O}_{2}max$, for most food groups a tendency toward higher CRF among participants with high intake could be observed. The food frequency questionnaire used in DEGS1 included a limited number of food groups of which some are relatively broad. Therefore, we did not adjust for overall energy intake^29^. Thus, higher CRF among participants with high intake of any food- and beverage group could be related to a higher energy requirement. However, the inclusion of physical activity as well as body mass index may partly adjust for energy needs.

### Socioeconomic and interpersonal factors

In the multivariable analyses, fitness was not associated with education or income, but we observed considerably higher fitness among women with high occupational status. While a previous study found that for other health indicators (e.g., smoking and obesity), education showed stronger effect sizes than occupational status, this was not the case for PA^61^. Other studies showed mixed results regarding the association between CRF and education, with a tendency for higher fitness levels among the highly educated^16^. A meta-analysis of four population-based studies (including DEGS1) found a positive association between education and CRF, but no relation after adjustment for PA^16^. While this meta-analysis adjusted for important confounders, no other measures of SES, such as occupational status or income were included. This may explain the differences with the results found in our study.

Higher fitness among individuals with high occupational status is in line with previous research^16^, although studies investigating the effect of occupational status on fitness are scarce. It is possible that lower occupational status is associated with higher levels of occupational PA^62,63^. Described as the ‘physical activity paradox’^64^, recent research suggests that there are no positive health effects of occupational PA. In fact, the effects of occupational PA might be inverse^65–67^. One hypothesized explanation for this paradox is that occupational PA is usually of too low intensity or too long duration without recovery time to improve CRF^68^. In addition, individuals with high occupational status tend to be more active during leisure time, improving their CRF^61,69,70^.

We found no evidence that interpersonal factors (social support and marital status) are strongly correlated with individual fitness. Overall, research on this topic is scarce. To our knowledge, there is no study that has investigated this association of social support with CRF so far. Regarding the relation of social support and PA, there is inconclusive evidence that social support is higher among more active individuals^12,71^.

Marital status was not considerably associated with CRF in our analysis, but, in contrast to women, divorced men tended to have higher fitness on average than married men. A longitudinal study from the US found that changes in marital status influence fitness status in men and women differently, supporting our observations: among men, the transition to being married was associated with a decrease in $\dot{V}{O}_{2}max$, while being divorced was associated with a modest non-significant increase. In contrast, no clear patterns were observed among women^72^.

### Anthropometric factors

We observed strong associations between the anthropometric measures BMI and WC and $\dot{V}{O}_{2}max$. In fact, the anthropometric factors showed the largest association among all behavioral, interpersonal and socioeconomic factors investigated, with the exception of PA-related variables.

Consistent with the findings of other studies, women and men with overweight or obesity had lower $\dot{V}{O}_{2}max$ than individuals with a normal BMI^73–76^. Furthermore, our results indicated a higher CRF for underweight women, but no relation between underweight and $\dot{V}{O}_{2}max$ was observed in men. Compared with the large number of studies that have investigated the association between continuous BMI or overweight or obesity (as measured by BMI), and CRF^8^, we are aware of only one study examining the association between underweight (defined by BMI) and CRF in adults. The study, conducted in a population-based sample from Taiwan reported lower CRF in underweight men, but not in women^77^. The strong relation between $\dot{V}{O}_{2}max$ and BMI may be generated by the definition of $\dot{V}{O}_{2}max$ as being relative to body weight^75^. Nevertheless, a study investigating $\dot{V}{O}_{2}max$ relative to fat-free mass also showed a negative association with obesity, as measured by BMI, in both men and women^78^.

Independent of BMI, increased WC was strongly associated with lower CRF in men and women. This is in line with previous findings investigating the association between abdominal obesity measured by WC and CRF^8,79,80^. It has been hypothesized that for specific health outcomes, a low CRF attenuates the health risk of obesity as measured by BMI^81^. Simultaneously, studies have shown that higher CRF is associated with less abdominal fat and visceral adipose tissue^82^. Thus, it can be argued that the larger health effects of CRF compared to BMI may be mediated by the reduced abdominal adiposity in individuals with higher fitness levels^82^.

### Physical activity-related factors

We observed strong associations between physical exercise as well as total PA and CRF among men and between physical exercise and CRF among women. It is empirically well documented that most people respond to regular physical exercise and training with short- and long-term physiological adaptations, which improve the CRF^83,84^. Greater activity amounts and intensities result, in general, in greater improvement of CRF^7^. Our results confirm this dose-response relationship with further increases of CRF with higher amounts of physical exercise per week. However, not all types of PA have the same beneficial effects for CRF, which could explain the differences for total PA compared with physical exercise found in our study. For example, occupational PA might be either of too low intensity or of too long duration to improve CRF. This might be the reason why total PA showed smaller effects sizes than physical exercise^67,85^.

### Practical implications

In Germany, there is great potential to increase the CRF of the general population^11,86^. The results of our study provide evidence to tailor interventions or prevention measures according to the needs of specific subgroups. For example, women with a low occupational position should be enabled to perform sufficient physical exercise to enhance their fitness levels. The suggested measures of the Global Action Plan on Physical Activity by the World Health Organization^87^ can be a good reference when planning measures to enhance the activity level of the population. Following the recommendations of the WHO, such measures should not solely focus on the individual, but also address the environment. In the case of women with low occupational status, this can for example translate into support for active transport to work or political measures to reconcile work and family life to enable more time for recreational PA. Furthermore, the association of $\dot{V}{O}_{2}max$ and consumption of specific foods might be an indication that different favorable health behaviors should not necessarily be seen separately, but rather be addressed at the same time. Again, such measures should focus on improvements of the living environment to foster individuals to make healthy choices.

### Strengths and limitations

Strengths of this study include the large population-based sample and its comprehensive nature, allowing for the investigation of a broad range of behavioral, interpersonal, and socioeconomic factors as potential correlates of CRF. Nonetheless, due to the cross-sectional design of the present study, no conclusions regarding causality can be drawn and there may have been potential bias related to reverse causality. The study sample consisted of a relatively healthy population that was rated as being test-qualified according to the PAR-Q screener, which could compromise the generalizability of the results. Another strength of the present study is that the measurement of CRF is based on a highly standardized and quality assured survey procedure^20,88^. In this study, as in most epidemiological studies investigating large populations^7,8^, we did not assess $\dot{V}{O}_{2}max$ directly via breath gas analyses, but estimated $\,\dot{V}{O}_{2}max$ based on a submaximal ergometer test. However, previous validation studies have shown that directly measured $\dot{V}{O}_{2}max$ in a maximal test and estimated $\dot{V}{O}_{2}max$ in a submaximal test are highly correlated^89^. Furthermore, the exposure variable physical exercise included information about the weekly duration but not about intensity which can have great impact on CRF^7^. Even though DEGS1 includes a wide range of health-related variables, some known correlates of CRF which were investigated in previous studies, e.g. caffeine consumption^90^, were not considered due to lacking information in the DEGS1 data set. Major efforts during the study process were made to reduce potential sources of bias^19^. Nevertheless, as most of the covariates were based on self-reporting by participants, reporting bias cannot be ruled out. Despite the various measures that were taken to enhance the willingness to participate, to account for unequal sampling probabilities and to adjust the distribution of the sample to the official population statistics, it cannot be ruled out that the relatively low response rate could have contributed to a potential selection bias. Although we used weighting factors, specific population groups, such as those with lower education status and individuals with migration background, may be underrepresented in our study.

## Conclusions

Despite the cross-sectional nature of the current study, we conclude that several factors at different domains of the conceptual framework are associated with CRF in the general adult population in Germany. These results can provide evidence to tailor prevention measures according to the needs of specific subgroups. Such measures should not solely focus on the individual, but also include actions on the environmental and political level.

## Supplementary information

Supplementary Information

## Data Availability

Datasets of DEGS1 are available as Public Use File: https://www.rki.de/EN/Content/Health_Monitoring/Public_Use_Files/application/application_node.html.
