# Supplementary material for: Individual and interpersonal correlates of cardiorespiratory fitness in adults – Findings from the German Health Interview and Examination Survey
Source: Sci Rep. 2020 Jan 16;10:445. doi: 10.1038/s41598-019-56698-z (PMC6965149; doi:10.1038/s41598-019-56698-z)
Supplement: Supplementary file 1 — Supplementary Information [file 41598_2019_56698_MOESM1_ESM.pdf]

Individual and interpersonal correlates of cardiorespiratory fitness in adults –
Findings from the German Health Interview and Examination Survey

Johannes Zeiher[a]*, Kristin Manz[a], Benjamin Kuntz[a], Nita Perumal[b], Thomas Keil[c,d,e], Gert B. M. Mensink[a], Jonas D. Finger[a]

[a] Department of Epidemiology and Health Monitoring, Robert Koch Institute, Berlin, Germany

[b] Department of Infectious Disease Epidemiology, Robert Koch Institute, Berlin, Germany

[c] Institute for Clinical Epidemiology and Biometry, University of Würzburg, Würzburg, Germany

[d] Institute for Health Resort Medicine and Health Promotion, Bavarian Health and Food Safety Authority, Bad Kissingen, Germany

[e] Institute for Social Medicine, Epidemiology and Health Economics, Charité - Universitätsmedizin Berlin, Berlin, Germany

*zeiherj@rki.de

Additional file 1: Supplementary figures and tables

Supplementary Table 1: Selected characteristics of study sample by test participation status

	Test participants		Test unqualified[a]		Total	
	%	(95%-CI)	%	(95%-CI)	%	(95%-CI)
**Sex**						
Men	52.6	(50.3-54.8)	46.2	(42.8-49.7)	50.3	(48.4-52.2)
Women	47.4	(45.2-49.7)	53.8	(50.3-57.2)	49.7	(47.8-51.6)
**Age**						
18-24 Years	16.7	(15.3-18.1)	5.9	(4.9-7.2)	12.8	(11.9-13.8)
25-34 Years	23.9	(22.0-25.9)	9.2	(7.5-11.4)	18.7	(17.2-20.3)
35-44 Years	24.7	(22.8-26.7)	18	(16.0-20.2)	22.3	(20.9-23.7)
45-54 Years	22.9	(21.2-24.7)	31.8	(29.3-34.5)	26.1	(24.7-27.5)
55-64 Years	11.8	(10.6-13.3)	35.0	(32.6-37.4)	20.1	(18.8-21.5)
**Retirement status**						
Not retired	97.2	(96.4-97.8)	87.4	(85.5-89.0)	93.7	(92.9-94.5)
Retired	2.8	(2.2-3.6)	12.6	(11.0-14.5)	6.3	(5.5-7.1)
**Born outside Germany**						
Born in Germany	85.3	(82.8-87.5)	86.1	(83.3-88.6)	85.6	(83.5-87.4)
Born outside Germany	14.7	(12.5-17.2)	13.9	(11.4-16.7)	14.4	(12.6-16.5)
**Educational Classification**						
Primary	24.9	(22.5-27.5)	40.4	(36.8-44.1)	30.4	(28.2-32.8)
Secondary	58.0	(55.4-60.5)	47.8	(44.5-51.2)	54.4	(52.2-56.5)
Tertiary	17.1	(14.9-19.6)	11.8	(10.1-13.7)	15.2	(13.5-17.1)
**Body mass index**						
Underweight (BMI <18.5)	1.9	(1.4-2.6)	1.0	(0.6-1.7)	1.6	(1.2-2.1)
Normal Weight (18.5 <=BMI <25)	49.4	(47.0-51.8)	31.7	(29.0-34.5)	43.1	(41.2-45.0)
Overweight (25 <=BMI <30)	35.3	(33.2-37.5)	33.0	(30.3-35.8)	34.5	(32.9-36.1)
Obese (BMI >=30)	13.4	(11.8-15.1)	34.3	(31.6-37.1)	20.9	(19.2-22.6)
**Physical activity per week**						
<2.5 hours	76.5	(74.4-78.4)	81.0	(78.7-83.1)	78.1	(76.5-79.6)
>=2.5 to <5 hours	13.7	(12.2-15.5)	9.0	(7.6-10.7)	12.1	(10.9-13.3)
>=5 hours	9.8	(8.5-11.2)	10.0	(8.5-11.8)	9.9	(8.8-11.0)
**Physical exercise per week**						
no physical exercise	42.8	(40.4-45.4)	57.4	(54.4-60.4)	48.0	(46.1-49.9)
< 2 hours	44.0	(41.7-46.4)	36.2	(33.4-39.1)	41.2	(39.5-43.0)
≥2 hours	13.1	(11.6-14.8)	6.4	(5.0-8.1)	10.7	(9.6-11.9)

[a] according to the Physical Activity Readiness Questionnaire
CI: confidence intervals

Supplementary Table 2: Correlates of $\dot{V}O_2max$ (full sample)

	Model 4	
	β	95 %-CI
**Sex**		
Men	(ref.)	
Women	**-6.56**	**(-7.17 - -5.94)**
**Age**		
18-25 Years	(ref.)	
25-34 Years	0.68	(-0.29 - 1.66)
35-44 Years	-0.20	(-1.30 - 0.91)
45-54 Years	**-1.49**	**(-2.61 - -0.37)**
55-64 Years	**-2.86**	**(-4.18 - -1.55)**
**Smoking status**		
Never	(ref.)	
Daily/Occasionally	0.29	(-0.38 - 0.95)
Former	-0.58	(-1.29 - 0.13)
**Alcohol consumption**		
Low	(ref.)	
Moderate	**0.78**	**(0.082 - 1.48)**
High	**1.49**	**(0.57 - 2.42)**
**Sugar-rich foods intake**		
Low/moderate	(ref.)	
High	**0.62**	**(0.01 - 1.23)**
**Sugar-rich drinks intake**		
Low/moderate	(ref.)	
High	-0.20	(-0.77 - 0.36)
**Junk foods intake**		
Low/moderate	(ref.)	
High	0.48	(-0.12 - 1.08)
**Fruit intake**		
Low/moderate	(ref.)	
High	**1.21**	**(0.65 - 1.76)**
**Vegetable intake**		
Low/moderate	(ref.)	
High	0.46	(-0.092 - 1.00)
**Place of birth**		
Born in Germany	(ref.)	
Born outside Germany	**-1.25**	**(-2.20 - -0.30)**
**Education**		
Primary	(ref.)	
Secondary	0.52	(-0.21 - 1.25)
Tertiary	0.36	(-0.78 - 1.50)
**Occupational status**		
Low	(ref.)	
Medium	0.38	(-0.41 - 1.18)
High	0.89	(-0.21 - 1.98)
**Income (% of median income)**		
<60 %	(ref.)	
60 to <150%	0.08	(-0.86 - 1.02)
>=150%	0.72	(-0.44 - 1.87)
**Social support**		
Poor	(ref.)	
Moderate	-0.57	(-1.54 - 0.39)
Strong	0.04	(-0.96 - 1.04)
**Marital status**		
Married, living together	(ref.)	
Single	-0.37	(-1.27 - 0.53)
Separated/Divorced/Widowed	0.33	(-0.76 - 1.42)

Supplementary Table 2: Continued

**Waist circumference**		
Normal	(ref.)	
Increased	**-1.58**	**(-2.31 - -0.86)**
Strongly increased	**-2.31**	**(-3.18 - -1.44)**
**Body mass index**		
Underweight	**2.23**	**(0.05 - 4.42)**
Normal Weight	(ref.)	
Overweight	**-2.68**	**(-3.37 - -1.99)**
Obese	**-5.42**	**(-6.48 - -4.35)**
**Physical activity per week**		
< 2.5 hours	(ref.)	
≥ 2.5 hours	**1.54**	**(0.77 - 2.30)**
**Physical exercise per week**		
No physical exercise	(ref.)	
< 2 hours	**1.83**	**(1.18 - 2.48)**
≥ 2 hours	**3.91**	**(3.08 - 4.74)**
**Constant**	**35.1**	**(33.0 - 37.1)**
**N**	2,826	
**R^2**	43,6%	

Coefficients and 95 %-CI and shown in bold: 95 %-CI does not include 0.
$\dot{V}O_2max$: maximal oxygen consumption; β: linear regression coefficient; CI: confidence intervals

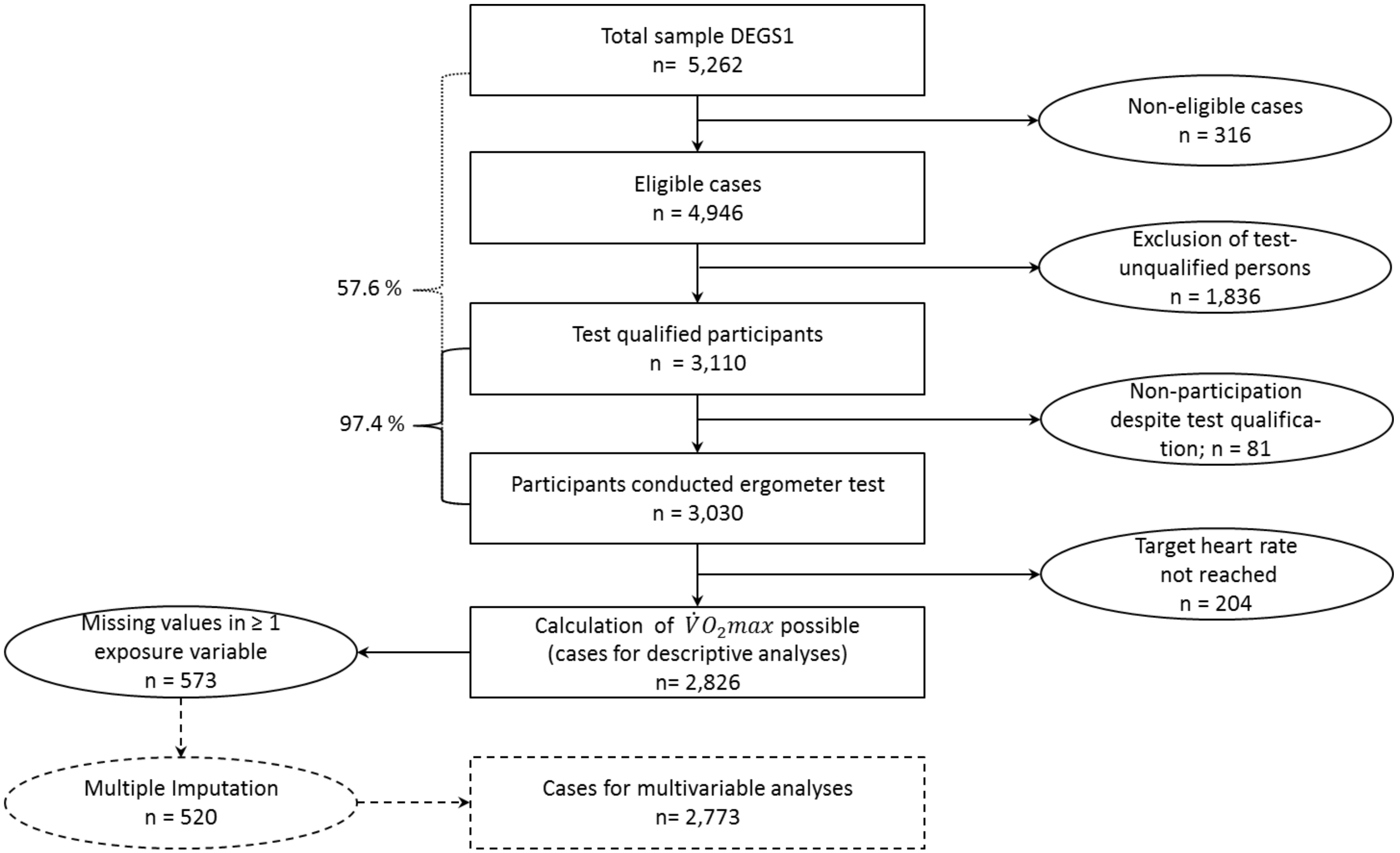

Supplementary Figure 1: Flow diagram of participants. DEGS1: German National Health Interview and Examination Survey for adults; $\dot{V}O_2max$: Maximal oxygen consumption

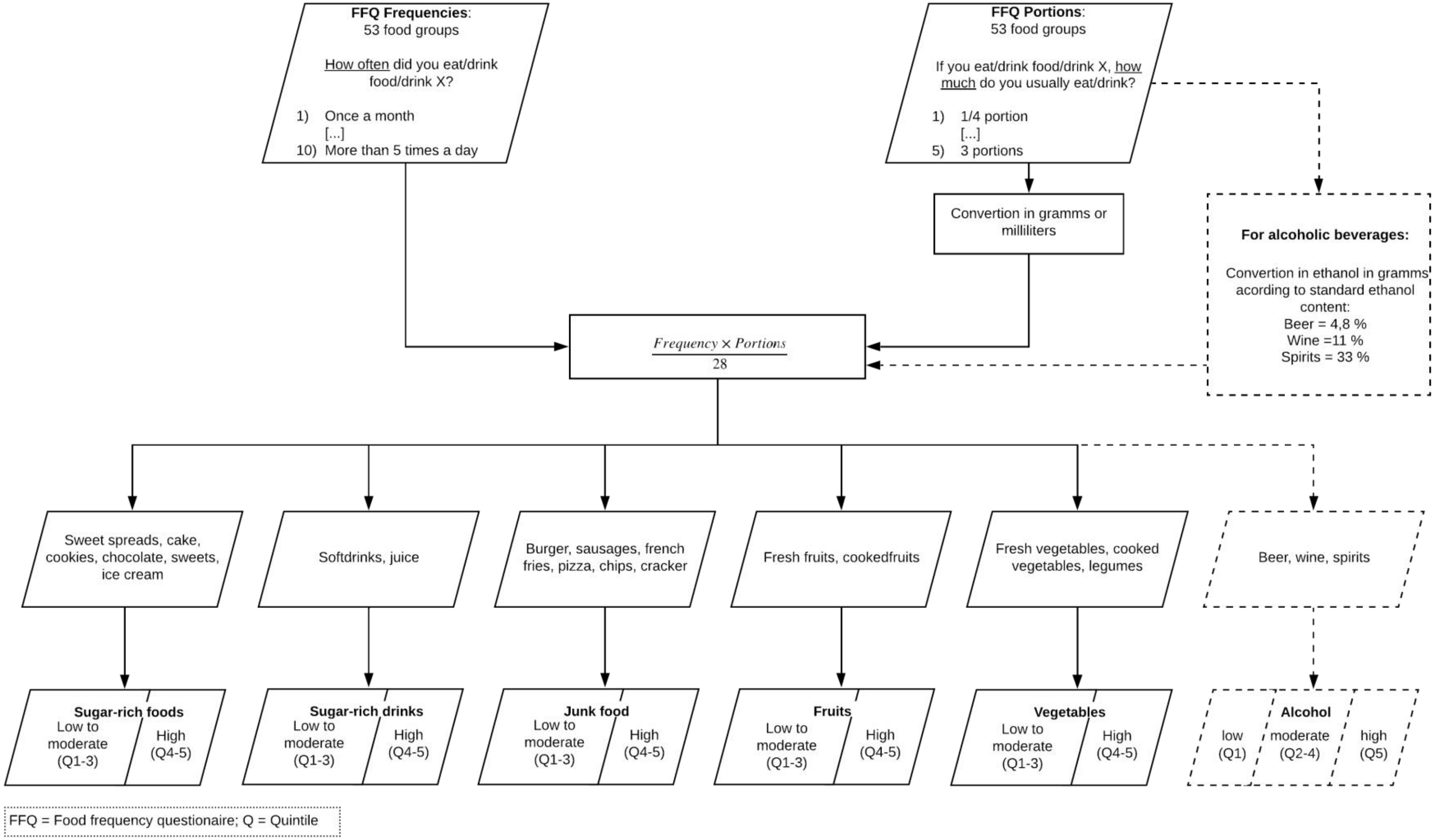

Supplementary Figure 2: Flowchart of food variable generation

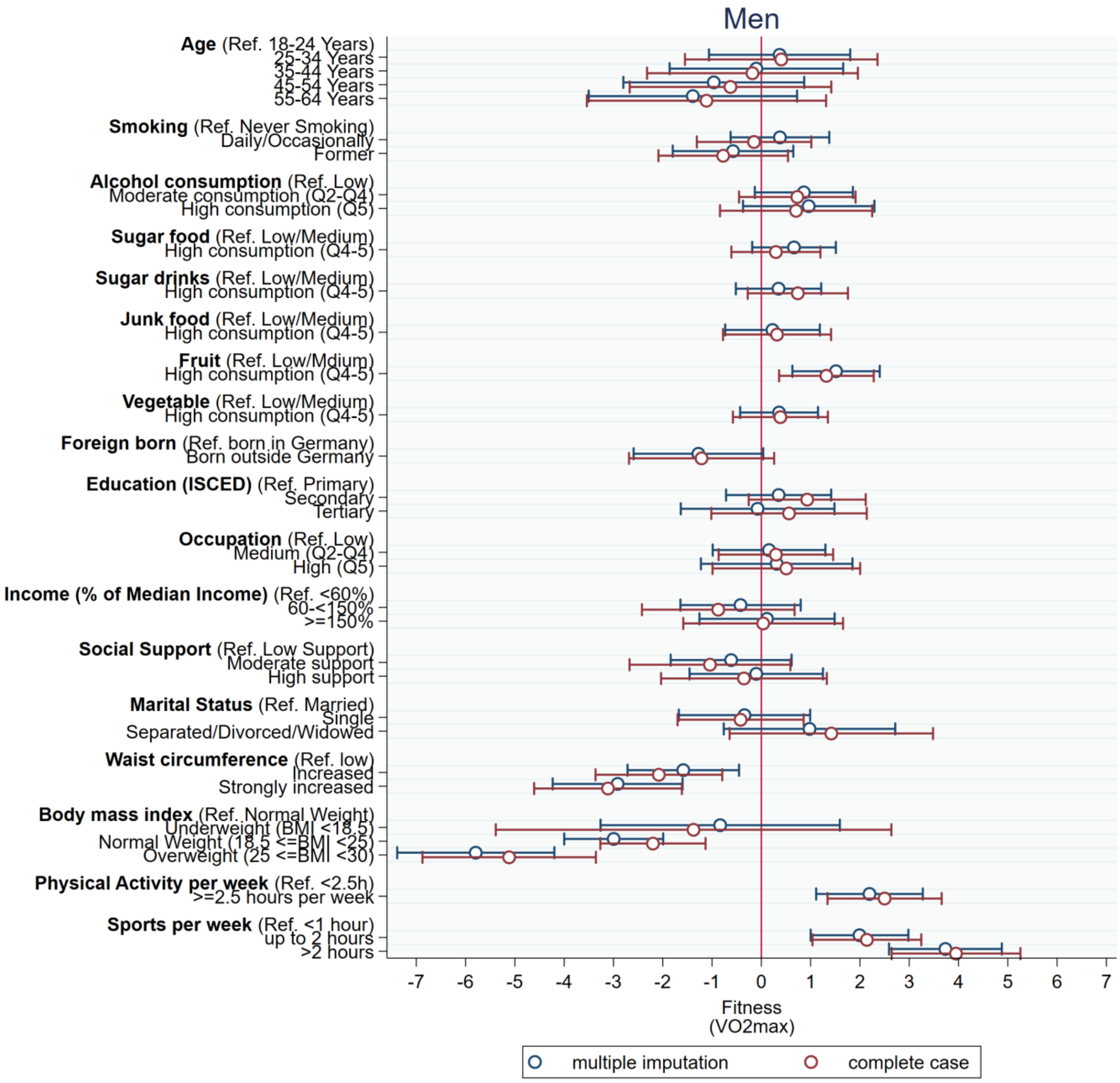

Supplementary Figure 3: Correlates of $\dot{V}O_2max$ in men. Comparison of an imputed model 4 with a complete case model 4 (fully adjusted).

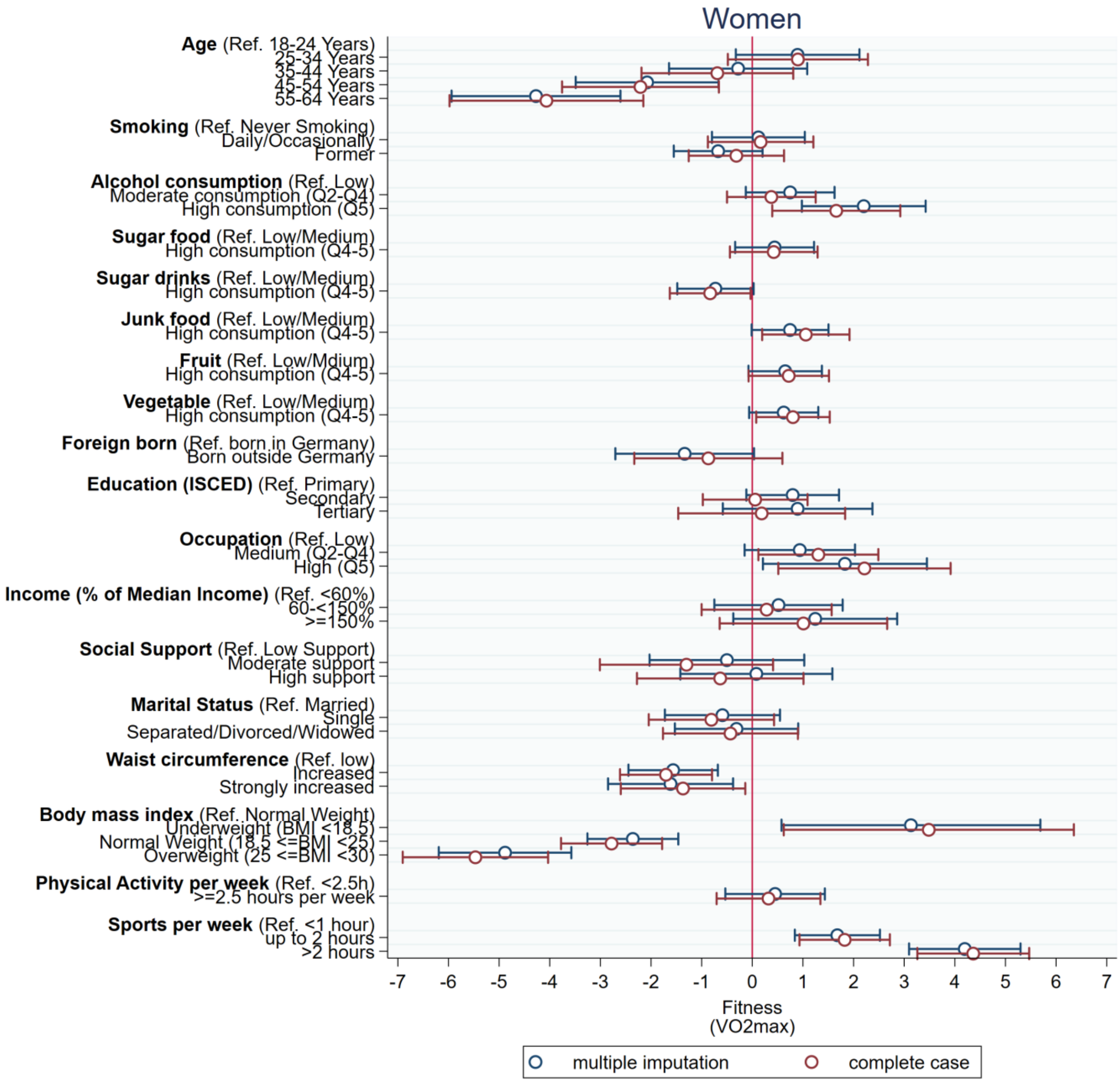

Supplementary Figure 4: Correlates of $\dot{V}O_2max$ in women. Comparison of an imputed model 4 with a complete case model 4 (fully adjusted).
